# Dengue: Moving from Current Standard of Care to State-of-the-Art Treatment

**DOI:** 10.1007/s40506-014-0025-1

**Published:** 2014-07-17

**Authors:** Victor C. Gan

**Affiliations:** Institute of Infectious Disease and Epidemiology, Tan Tock Seng Hospital, 11 Jalan Tan Tock Seng, Singapore, 308433 Singapore

**Keywords:** Dengue, Dengue hemorrhagic fever, Dengue shock syndrome, Warning signs, Severe dengue

## Abstract

Treatment of dengue remains supportive in the absence of targeted antiviral therapy or approved vaccines. Responsive fluid management is key to preventing progression to shock or other severe manifestations. The dynamic natural history of dengue infection and its influence on hemodynamic homeostasis needs to be carefully considered in the planning of individualized therapy. Though largely self-limiting, the sheer burden of dengue disease on the global population will result in atypical manifestations especially in children, older adults, and comorbid patients. Management of these has not yet been systematized. The failure of recent randomized controlled trials to show utility for antiviral and immunomodulatory agents in dengue is disappointing. Vaccine candidates hold promise, but growing outbreaks require more robust, evidence-based management guidelines to inform clinicians, especially in novel epidemic situations.

## Introduction

Dengue is caused by a flavivirus comprising four established serotypes (DENV1–4), with a fifth serotype recently proposed [1]. Dengue has the greatest reach of all arthropod-borne viral (arboviral) infections, with an estimated 390 million infections a year [2]. Dengue infection manifests with a variety of clinical syndromes, from asymptomatic infection, to undifferentiated acute febrile illness, to dengue hemorrhagic fever and severe dengue, including dengue shock syndrome [3•]. Historically, dengue has been one of a number of mosquito-borne acute febrile illnesses, and differentiation from other flaviviruses such as yellow fever, alphaviruses such as chikungunya, or parasitic infections such as malaria has not always been possible when attributing etiologic agents to historic outbreaks from the late 18th century until the isolation of dengue virus in 1943 [4, 5].

One of the earliest accounts of what was then known as breakbone fever or Dandy fever was by Benjamin Rush, describing the outbreak in Philadelphia in 1780 [6]. He already recognized that the vast majority of patients recover without major complications after suffering for a few days of fevers and aches. He recommended bed rest (rather than exercise), and treated patients with emetics and laxatives following the practice of the day, along with opiates. He recognized the occasional complication from bleeding and warned against blood letting, otherwise a popular recourse. Similar treatment for this largely non-fatal fever continued to be recommended by physicians in the outbreaks of the early 19th century, for example in India [7], Zanzibar (Tanzania) [8], and South Carolina [9]. As one of many tropical fevers, generic folk remedies recorded historically and currently in vogue included porcupine bezoars [10–12] and papaya leaf extract. Several publications examining the latter in the context of dengue infection have given equivocal results [13–16]. Into the early 20th century, treatment has remained supportive, primarily comprising analgesics and antipyretics for symptomatic relief, with the management of complications as they arise, such as bleeding, dehydration, shock, or organ failure.

The impact of dengue precipitously increased after World War II where a host of epidemiologic and ecologic factors affecting the spread of Aedes aegypti, urbanization trends, and increased global travel led to large pediatric epidemics with high mortality in southeast Asia [17] and later in the Americas [5]. The clinical experience derived from these epidemics allowed physicians to develop clinical protocols to reduce mortality, which were promulgated by the World Health Organization (WHO) [18]. The key intervention was understood to be intensive intravenous fluid administration to counteract the fluid leak from capillaries in the critical defervescent phase, when children in particular are prone to develop shock and organ failure. Development of the concept of dengue hemorrhagic fever as a syndromic precursor to shock and death led to an emphasis on identifying candidates for judicious rehydration by frequent monitoring of blood pressure, haematocrit, and signs of shock such as capillary refill and other signs of peripheral circulatory collapse. Implementation of standardized treatment guidelines led to a marked reduction in case fatality from over 13 % to less than 0.1 % in Thailand [19]. Increasing adherence to guidelines (primarily encouraging oral rather than intravenous fluid therapy where appropriate) has been shown to lead to gains in resource use in dengue case management without adversely affecting morbidity or mortality in Nicaragua [20]. The importance of optimizing guidelines locally has also been shown in Singapore, where significant cost savings were associated with reductions in unnecessary hospitalization after admission criteria were modified and more strictly enforced [21].

## Treatment guidelines

Dengue management algorithms have been developed by the WHO South East Asia Regional Organization (SEARO) and the WHO Pan American Health Organization, which have been further adapted in national guidelines. The premise is distinction of dengue disease from other tropical fevers, for which clinical criteria alone are not sufficiently discriminatory, especially in older adults [22–24]. Diagnostic testing is strongly recommended, summarized in Table 1. In brief, in endemic areas or in likely immune populations with past exposure to dengue or other related flaviviruses, reverse transcription-polymerase chain reaction (RT-PCR) and non-structural protein 1 (NS1) detection are most useful because of their specificity, particularly in early presentation. In late presentation, clinical manifestations may have to guide management with a battery of repeated tests requiring evidence synthesis by experienced physicians to determine etiology. In travelers or other non-immune populations, in addition to RT-PCR and NS1 in the early or febrile phase, immunoglobulin M and immunoglobulin G may be more useful, but with caveats about accuracy. In the context of an acute undifferentiated tropical fever otherwise negative for common etiologic agents such as *Plasmodium* or *Salmonella*, the combination of leukopenia, severe thrombocytopenia (<50 000/mm^3^), rapid changes in hematocrit (>20 % change from baseline or initial value), and elevated transaminases with an asparatate transaminase:alanine transaminase ratio of >1 make dengue the prime candidate. Mild cases that do not reach these values are likely self-limiting or may not benefit from currently available treatment.

**Table 1:** Dengue diagnostic testing

Diagnostic Modality	Sample and Processing Required	Indications	Advantages	Limitations	Future Developments
Viral RNA detection through reverse-transcription polymerase chain reaction (RT-PCR)	Serum and other clinical samples (e.g., CSF, tissue, urine [25]). Laboratory required	For early detection during viremic phase (typically while febrile, decreasing sensitivity over first 3–5 days of fever). Negative results may not rule out dengue due to exponential decrease in viral loads within 48–72 h of fever	Able to distinguish from other flaviviruses and to serotype DENV1–4. Potentially very sensitive and specific	Often negative during late (severe) clinical presentation. Sensitive to laboratory procedures and requires stringent quality control [26]. Longer turnaround time. Often only done in regional reference laboratories. High cost	Faster, more accurate, robust methods of nucleic acid (NA) detection in development. Use of NA quantitation as surrogate for antiviral effectiveness being trialled
Viral non-structural protein 1 (NS1) antigen detection	Serum, urine [27], CSF [28], oral swabs [29]. Rapid immuno-chromatographic testing (ICT) or ELISA-based laboratory testing	For detection during the symptomatic phase, with longer period of detection than PCR (antigenemia often still present at discharge from care [30]). May have lower sensitivity on day 1 [31] (not clinically critical)	Best current option for point-of-care testing [32, 33]. Combination with antibody detection leads to high accuracy [32, 34, 35]	Relative high cost of individual sample ICT. Need for laboratory for ELISA-based testing. Sensitivity may be reduced in secondary dengue and in DENV-4 [36, 37]. Specificity for widespread use in hospital populations with comorbidities (e.g., rheumatologic conditions) not well evaluated	Improved accuracy and robustness in development. Serotype-specific NS1 testing in research use [38, 39]
Antibody detection (IgM)	Serum, CSF and other tissues. Rapid ICT and ELISA-based laboratory testing	Elevated in primary infection starting typically 5–7 days into illness. Often detectable only in the defervescent or even convalescent phase. Repeated testing for seroconversion should be done	Low cost. May contribute to diagnostic battery when direct detection is negative. Most useful in known non-immunes (e.g., travelers).	May be confounded by intercurrent infection (e.g., malaria), non-specific boosting of previous exposure to dengue or related flaviviruses (including vaccine exposure) [40, 41]. Rapid tests “did not show acceptable performance” in a WHO-TDR evaluation [40]	May continue to be used as component in multiple testing. Not ideal as a single acute test
Antibody detection (IgG)	Serum, CSF, and other tissues. Rapid ICT and ELISA-based laboratory testing	Most useful as marker of past infection rather than acute infection. Markedly high levels may indicate secondary infection but should not be the only criterion used for diagnosis. Seroconversion is more reliable than a single acute reading to indicate acute infection	Low cost. Used to determine primary/secondary status. May be the only positive test in secondary cases presenting late in illness	May be confounded by intercurrent infection or previous exposure to related flaviviruses (including vaccine exposure). Threshold values for levels indicating secondary infection not well established	Increased accuracy required for reliable determination of past infection. Lack of clinical demand as not critical for diagnosis of acute dengue
Antibody detection (IgA)	Serum, saliva [42]. Rapid ICT and ELISA-based laboratory testing	Similar profile to IgM but may be more sensitive in secondary cases [43]	Potential improvement on IgM	Relatively new test. Requires extensive research and field trials to evaluate utility. Not widely available	Potential for non-invasive sampling from saliva promising. Commercial incentive to improve IgA-based testing is low
Plaque neutralization antibody test	Serum. Virus-producing laboratory and experienced personnel required	Classic definitive test for dengue-specific protective antibodies. Determination of previous infection as well as immunologic progress in acute infection using repeated samples. Not used routinely for clinical purposes	Extensive historical experience in use. Quantitation established for levels in acute infection. Serotype-specific response determination possible. Least prone to cross-reactivity with other flaviviruses	Resource intensive to perform. Correlation to immunologic protection increasingly called into question, particularly considering serotype-specific responses [44]. Virus strain dependent	Maintained in reference laboratories and vaccine trials as “gold standard” [45]. Vaccine trials moving towards use of engineered DENV reporter virus particle in a high-throughput flow-cytometric system to measure neutralization [46]
Viral isolation	Serum, tissues	Research test for clinical strain propagation and phenotypic charaterization. Not as sensitive as PCR. Will not give a result within timeframe required for clinical management	Critical for definitive identification and continued characterization of clinical strains	Resource intensive. Seldom useful clinically. Best pickup at high viremia prior to fever onset	Sequencing and reverse genetics techniques may encroach on the need for isolation

*CSF* cerebrospinal fluid, *ELISA* enzyme-linked immunosorbent assay, *IgA* immunoglobulin A, *IgG* immunoglobulin G, *IgM* immunoglobulin M, *WHO*-*TDR* World Health Organization-Tropical Diseases Research

**Table 2:** Fluid management in dengue dependent on effect of capillary leak on hemodynamic status

	Initial Determination [47, 52]	Main Management Decisions	Issues for Consideration	Outcomes/sequelae
Mild dehydration	Clinically stable, hemodynamic parameters and hematocrit within normal range	Monitor clinical status and blood counts at least daily and watch for change in condition with defervescence, usually 5–7 days after fever initiation [53••]. Deterioration may be sudden	Concomitant pathologies or comorbidities complicating progress especially in an epidemic situation with a large proportion of population infected [59] .Atypical manifestations are rare and generally self-limiting [60]	Rise in platelet count indicates transition to convalescent phase. Symptomatic improvement precedes resolution of cytopenias and transaminitis. Sequelae other than fatigue rare in dengue
Significant dehydration at risk of shock or compensated shock	Changes in hematocrit from baseline or initial determination of >20 % indicate significant capillary leak. Clinical examination or imaging may reveal pleural effusion or ascites. Deterioration of hemodynamic status such as tachycardia, dropping blood pressure, or decreasing pulse pressure prompt close attention	Judicious hydration critical. maintenance IV fluid should be given to blunt or reverse hematocrit rise with strict recording of fluid balance to prevent overhydration [48•]. Frequent monitoring and review of fluid administration rates several times a day should be performed. During transition to critical (defervescent) phase, IV hydration may need to be increased as hemoconcentration accelerates but this period should last 24–48 h, and subsequent reduction of fluid administration is vital as fluid shifts from third spaces back into the vascular system with convalescence [61]	Watch for occult bleeding clinically as well as monitoring blood counts, though simultaneous plasma leakage, IV fluid administration and bleeding may make interpretation of changing hematocrit and hemoglobin levels difficult. Watch for atypical organ manifestations of dengue such as encephalopathy or cardiac abnormalities and ensure secondary causes fully investigated. Underhydration or overhydration both potentially dangerous	Judicious hydration can prevent shock or organ damage. Recovery may be prolonged but improvement in all parameters should commence and persist from 2 to 3 days after defervescence, failing which further investigation for other etiologies should be pursued
Decompensated shock due to fluid leakage	Hemodynamic parameters compromised, with hypotension, pulse pressure <10 mmHg, signs of peripheral circulatory collapse. Occurs during critical phase, typically 5–7 days into illness	Intensive care required with careful monitoring of fluid status. Primary intervention is IV fluid, initially crystalloid, with the use of colloids if response poor [62]. All organ systems may be compromised so pre-emptive monitoring may be helpful	Children, older adults [63, 64], and comorbid patients at particular risk. Intensive care protocols for hydration in septic shock may lead to over-hydation if dynamic natural history of dengue is not taken into account. Driving fluid into interstitial space can impair organ function or overload the cardiovascular system when reabsorption occurs in convalescence [65]. Close interaction between clinical specialists (e.g., intensivists and infectious disease physicians) paramount	Organ impairment may lead to secondary disease manifestations that require separate management, such as renal replacement therapy. Hospital acquired infection may complicate course [63]. Immunological sequelae not well described in those with extensive comorbidities. Fatal outcome sometimes unpredictable, even in healthy young adults

*IV* intravenous

There is significant variation between the revised WHO 1997 guidelines [47], regional guidelines [48•, 49], and national guidelines [50]. Subsequently, recognizing the limitations in the sensitivity of depending solely on a strict adherence to dengue hemorrhagic fever criteria (fever, thrombocytopenia, capillary leak, and hemorrhagic tendency) to determine treatment prioritization [51], WHO proposed new dengue severity classifications and adjusted management algorithms in 2009 [52] and expanded treatment guidelines in 2012 [53••]. While the key principles of timely and judicious fluid repletion are maintained, different criteria have been proposed for triage, hospital admission, and intensive care unit admission, as well as initiation, rates, and cessation of intravenous fluid administration. Criticism of this new guideline centers around two aspects. First, progression to severe dengue is not considered as a single pathophysiologic entity but as a diverse set of manifestations, complicating the streamlining of management, especially by inexperienced staff, and hampering focused pathogenesis research [54]. Second, the broader criteria for clinical diagnosis of dengue and particularly dengue requiring hospitalization is regarded as potentially problematic, taking into account the lack of rapid definitive dengue laboratory diagnosis and lack of inpatient beds especially in outbreak situations [55–58].

## Fluid management

The primary modality for therapeutic intervention in dengue is fluid management. The fundamental principle building on experience in Thailand from the post-war period until now and articulated most clearly in the recent WHO SEARO guidelines [48•] is of dengue as a triphasic syndrome. In the initial febrile phase lasting 3–7 days, serum viremia is present, and increasing capillary permeability leads to a rise in hematocrit. This may be exacerbated by dehydration due to anorexia, nausea, vomiting, diarrhea, and malaise. Together with the development of a serologic response, and associated with defervescence, the critical phase is one where intervention can make the most difference. This is when immunopathologic responses are maximal, reflected in a platelet nadir and rapid rise in hematocrit as fluid shifts out of the intravascular space into the interstitium and third spaces. There is the greatest risk of organ dysfunction exacerbated by edema and microvascular changes during the critical phase, which lasts 1–2 days. The possibility of iatrogenic fluid overload through cumulative fluid administration is also greatest here. Subsequently, during the convalescent phase, blood dyscrasias normalize, inflammatory markers settle, and fluid shifts back into the vasculature. There is a risk of hemodynamic stress if the patient was in fluid overload and the shift of interstitial fluid overwhelms the capacity of the cardiovascular system at this point.

As this dynamic process unfolds, clinical fluid interventions are overlaid on this shifting landscape, making identification of the stage the patient is at the key to good management. Unfortunately, no simple algorithm is able to categorize patient response, given the wide range of variation of ages and fitness represented. Those at higher risk of being unable to cope with these fluid shifts as measured by changes in microvascular permeability (such as children, or those genetically predisposed) may be at higher risk of shock [65]. Clinical judgment taking into number of days since fever onset, body temperature, blood pressure, signs of peripheral circulatory status, and signs of fluid overload including radiographic and ultrasonographic studies is required.

Importantly, goal-directed therapy in dengue shock is different from that in septic shock as the physiologic and homeostatic context of the cardiovascular system is distinct in these two syndromes. Even in the fluid management of infection-associated shock, existing guidelines have been called into question by the FEAST trial that showed standard fluid boluses given in hypotension led to increased mortality in such pediatric cases [66]. In addition to goal-directed therapy using catheter-based blood pressure measurement and targets, a more sophisticated approach taking into account measures of tissue perfusion, fluid balance, and capillary leak may be required [67]. These may include such emerging non-invasive technologies such as photoplethysmography which uses changes in pulse oximetry waveforms to indicate changes in cardiovascular status [68], and is being studied in the context of dengue [69].

Dengue patients can be differentiated into three categories for the purposes of fluid management: mild dehydration; significant dehydration at risk of shock or in compensated shock; and decompensated shock due to fluid leakage. Patients may stay in the first category throughout their illness, or move into the second category just before and during the critical phase, or progress further into the third category during the critical phase. The time-dependent progression is important as, for example, aggressive fluid therapy for presumed compensated dengue shock if given early in the course of illness and for a prolonged period of time may lead to fluid overload Table 2 summarizes the key management decision points for clinicians, with selected issues discussed further below.

Treatment of mild dehydration is the mainstay of hospitalized dengue. Oral rehydration using isotonic electrolyte solutions or similar fluids such as coconut water or rice water are recommended in many guidelines. These carry less risk of iatrogenic fluid overload than intravenous administration and have been associated with decreased resource use while maintaining safe outcomes [20]. Anti-emetics may be useful to control vomiting. Mild hypokalemia is often encountered, likely associated with vomiting and diarrhea, but is seldom of sufficient magnitude to cause clinical significance and resolves in early convalescence.

Those in the second category may be identified by rapidly rising hematocrit, rapid drop in serum protein or albumin levels, or clinical fluid accumulation such as pulmonary edema and ascites. These are components of the definition of dengue hemorrhagic fever, which requires in addition signs of hemorrhage and thrombocytopenia. Whether the latter are essential to the assignment of at-risk status needing additional fluid administration has been controversial, with the latest WHO SEARO guidelines emphasizing capillary leak criteria over hemorrhagic criteria in determining fluid management [48•]. Determining numerical thresholds for these criteria is challenging given individual physiologic variation. Guidelines currently refer to relative changes in hematocrit over 10 % as a warning sign and changes over 20 % as a clear indication for more aggressive fluid therapy. One model for guiding fluid administration follows an inverted ‘V’ pattern over 48 h, rising from 40 mL/h to 200 mL/h and falling back to 40 mL/h in adults [48•]. Judicious fluid management requires frequent monitoring and sensitivity to clinical status. Isotonic crystalloid is universally recommended in uncomplicated cases except in infants where half-molar sodium chloride may be used [52]. It should be reiterated that the rates of intravenous fluid administration detailed above are envisioned to be used over a 24- to 48-h period, and subject to clinical monitoring as significant morbidity and mortality can result from fluid overload [70]. The WHO 2009 guidelines also suggest that warning signs are indications for an increased intravenous fluid administration rate over maintenance. There has been vigorous discussion regarding this as the list of warning signs are clinically varied and do not describe a single pathophysiologic syndrome [54], so it may be more appropriate to individualize fluid administration rates rather than group them together under one protocol.

By good fluid management at the transition to the critical phase, the aim is primary prevention of progression to shock. If dengue progresses into shock because of fluid leakage, management should be in an intensive care setting, with the ultimate goal to maintain tissue perfusion. Intensive care protocols such as surviving sepsis bundles will often be applied, but should be tempered by recognition of the different context of fluid shifts in dengue compared with septic shock. Protocols are given in WHO guidelines, including evidence from randomized controlled trials that initial treatment can begin with crystalloid infusions, with hyper-oncotic colloids as a second-line agent where response is not optimal [71, 72]. The ideal is to titrate intravenous fluids along the natural progression of dengue fluid leak, which increases then decreases sharply during the 24- to 48-h critical phase, to blunt the impact on tissue perfusion and hemodynamic homeostasis. The WHO SEARO 2011 guidelines highlight that obese patients should not be given fluids according to actual body weight but according to ideal body weight to avoid fluid overload.

## Antiviral and other targeted therapy

There are as yet no specific antiviral therapies for dengue. Active research is ongoing using in vitro and animal models [73], but few drugs have entered clinical trials. Chloroquine has been shown to be ineffective in reducing viremia [74] and disease duration but may offer limited symptomatic relief [75]. Dengue antiviral research has benefited from the recent intensive effort to discover drugs for the treatment of hepatitis C virus (HCV), a fellow flavivirus. A number of HCV drugs that have pre-clinical efficacy against dengue virus have been repurposed and moved on to phase II clinical trials for dengue. Balapiravir was shown to be safe in adults with dengue, but did not lead to changes in the kinetics of viremia or cytokine profiles [76]. Celgosivir, also a repurposed HCV candidate, has similarly shown a lack of success in reducing viral load or fever in patients with dengue [77]. Other candidates are in the drug discovery pipeline, which has been growing with increased global emphasis on neglected tropical diseases [78]. Finally, there is a potential for using dengue-specific antibodies therapeutically by neutralizing dengue virus [79]. The key hurdle to this is ensuring that antibody-dependent enhancement of DENV infection does not take place.

## Immunomodulatory agents

A major component of dengue disease is a result of immunopathology, for which the metaphor of a cytokine storm has been used. In the process of attempting to eliminate dengue virus, changes in physiology occur which, if transient, result in a good outcome after a period of acute morbidity, but in cases where the host physiology is not robust enough to weather the change (e.g., in children or older adults), irreversible damage may result. Though our understanding of the precise nature of the immune response against dengue is still in development, attempts have been made to trial immunomodulatory agents. Intravenous immunoglobulin has been shown to lack efficacy [80, 81]. Recent guidelines have emphasized the lack of evidence for using corticosteroids [82]. A small randomized controlled trial was conducted in Pakistan that showed a limited but statistically significant difference in platelet recovery between treatment and placebo groups using interleukin-11; however, no other clinically significant endpoints were used [83]. Finally, statins exhibit pleiotropic effects on the inflammatory response in humans [84, 85] and antiviral effects in mouse models of dengue [86]. A randomized controlled trial is in progress in Vietnam to assess the effect of lovastatin in dengue [87].

## Vaccine development

A dengue vaccine has been held out as the hope for disease control and eradication since 1929 when the first candidates were evaluated [88, 89]. To date, the Gates Foundation’s strategy for dengue is enhanced vaccine research rather than drug discovery. There has been a range of vaccine candidates proposed, using live attenuated virus, chimeric virus, inactivated virus, subunit vaccines, and DNA vaccines [90]. In addition to the usual concerns of safety and immunogenicity, dengue vaccines have been stymied by the prospect of antibody-dependent enhancement, a feature that risks worsening the effect of dengue infection through immunologic memory [91]. This results from the presence of four potentially cross-reactive serotypes, as well as the potential for cross-reactivity with related flaviviruses. All candidates have to be extensively investigated to ensure this theoretical risk is eliminated. There is currently only one candidate in phase III efficacy trials; however, the initial report from a phase IIb pediatric cohort in Thailand indicated poor vaccine efficacy against DENV-2 [92••]. The results of the full trial in Asia and Latin America are awaited eagerly.

## Systems-based management

### Systemic manifestations: pyrexia

The role of antipyresis in infectious fevers remains inconclusive with the aim being primarily symptomatic relief and the effect on clinical measures of microbiologic clearance, morbidity (e.g., febrile seizures), and mortality not well established [93, 94]. In dengue, the febrile phase generally lasts 3–7 days with an upper limit of 10 days in unusual cases. A biphasic (or saddleback) pattern has been reported in some cases with a transient defervescence around day 5 and subsequent progression to the critical phase associated with risk of shock and immunolopathologic manifestations. Limited research has been conducted on pharmacologic (paracetamol/acetaminophen) [95] and physical (sponging) methods [96] of antipyresis but both remain recommended in the WHO guidelines for dengue [52]. The utility for analgesia may contribute to the near-universal use of paracetamol in dengue. However, the inherent potential for liver toxicity as an adverse effect with paracetamol may be magnified by the tendency of dengue to cause liver inflammation as measured by elevated aminotransferase levels [97–99]. Non-steroidal anti-inflammatory drugs are contraindicated because of the danger of exacerbation of bleeding in the context of depressed platelet levels during acute dengue. For patients receiving long-term antiplatelet therapy with aspirin, clopidogrel, or similar agents, physicians may consider temporarily withholding these drugs until the thrombocytopenia during acute dengue improves, as the risk of thromboembolism during this period is reduced and risk of hemorrhage increased. Finally, corticosteroids have been shown in randomized controlled trials to have no significant impact on outcomes in severe dengue [82, 100–102]. Though not recommended in guidelines, there remain anecdotal reports of their use in symptomatic relief, though the complex immunopathology of dengue should give physicians reason for careful consideration before use. One important factor in the use of antipyretics for dengue is the critical dependence on the recognition of progression from the febrile phase to the critical (defervescent) phase with the concomitant shifts in fluids that take place during this progression. The amelioration of pyrexia can confuse determination of what phase the patient is in, and subsequently affect decisions on fluid management that depend on the shifting intravascular versus third-space fluid volume of the patient.

### Musculoskeletal manifestations

Aches and pains are of major clinical significance during dengue, being key symptoms that aid in clinical diagnosis and often are the primary concern of the patient. Bed rest and paracetamol remain the mainstays of management. Less commonly, opiates or even corticosteroids have been used [103]. Rhabdomyolysis, reflected in raised serum creatinine kinase is a recognized complication [104], which in rare cases can lead to acute kidney injury, recently confirmed in one case report with a renal biopsy [105]. In most mild cases, adequate hydration that follows the standard of care for dengue will help recovery. Progression to renal dysfunction though may necessitate hemodialysis. There may also be transient muscle dysfunction associated with muscle weakness and hyporeflexia that does not require treatment and is generally self-limiting [106].

### Dermatologic manifestations

Cutaneous changes in dengue can be polymorphic [107, 108]. The classical dengue rash occurs in two phases: in the early febrile period, generalized flushing occurs, sometimes manifesting as a morbiliform erythematous macular rash, followed in the late febrile and defervescent period by the typical maculopapular rash with “islands of sparing” alongside petechiae and ecchymoses associated with severe thrombocytopenia. In a small number of cases, recovery may be delayed by days to weeks after defervescence, with some residual macular erythema consistent with a delayed-type, T-cell-associated cutaneous reaction. Apart from patient anxiety from the cosmetic effect of the rash that can be alarming in its intensity and spread, the main treatable sequela is pruritis, which occurs in a large proportion of cases. Antihistamines can be used, where the adverse effect of drowsiness may fortuitously aid in supporting bed rest.

### Opthalmic manifestations

Generally, visual disturbances are rare in dengue, but a number of case series have been reported from epidemics in Singapore [109–112] and India [113, 114]. Symptoms include blurring of vision, subconjunctival hemorrhage, eye flashes, floaters, and retro-orbital pain. Pathologic causes identified include maculopathy, exudates, retinal hemorrhage, retinal detachment, vasculitis, foveitis, choroiditis, and uveitis. These largely occur during the critical (defervescent) phase of dengue and resolve in the convalescent phase, though persistence has been described in a few individuals. Serotype association has been proposed [115] as well as a relationship to autoimmune disease [116]. Full recovery usually occurs in the absence of specific treatment, though systemic and topical corticosteroids have been given with good results in a few cases.

### Neurological manifestations

A recent review of neurologic complications of dengue virus infection used the following categories: encephalopathy, encephalitis, and immune-mediated syndromes in addition to muscular and ophthalmic involvement already covered [117•]. Lack of biopsy findings often precludes definitive diagnosis, as does difficulty in proving dengue virus involvement, especially in regions endemic for cross-reactive flaviviruses where interpretation of serologic testing is not straightforward. Further, when large outbreaks occur that affect significant proportions of residents, Occam’s razor may not apply and acute confusional states may have multiple infective or other etiologies. Encephalopathy may be multifactorial, exacerbated by acute liver failure, metabolic derangements, shock, or intracranial hemorrhage. Dengue-associated encephalitis often does not display classical radiographic characteristics of viral encephalitides. These should be treated according to evidence-based guidelines to optimize outcomes. Otherwise, treatment for central nervous system complications of dengue remains supportive. Some anecdotal success has been reported using corticosteroids or intravenous immunoglobulin for immune-mediated complications [118, 119] but no randomized controlled trials have taken place.

### Hematologic manifestations

Neutropenia, lymphocytopenia, and thrombocytopenia are all cardinal signs of dengue virus infections that usually resolve within 1–2 weeks. Despite severe neutropenia with an absolute neutrophil count of less than 0.5 × 10^9^/L occurring in 12 % of one dengue cohort, it was not associated with poorer outcomes or an increase in secondary infections, and usually resolved within 1 day, thus there may not be a need for routine prophylactic antibiotics [120]. A single case report of the use of filgrastim as rescue therapy for persistent neutropenia in a case with multiple complications and delayed defervescence has been published [121]. The use of granulocyte-colony-stimulating factor has also been described in one Thai pediatric case [122]. Lymphopenia is often the first to resolve, with a rapid increase of reactive lymphocytes being the earliest sign occurring during the defervescent phase. No specific therapy has been described for this.

Thrombocytopenia is of the most concern because of the increased risk of hemorrhage. Minor mucosal bleeding is relatively common, particularly transient gingival bleeding or epistaxis, which may be treated with gentle pressure. Tranexamic acid, an anti-fibrinolytic agent, has been suggested in such cases, though it has not yet been formally evaluated in dengue [123, 124]. Excessive vaginal bleeding occurred in 27 % of female adolescents in one Thai study [125] who were treated with premarin, primulute N, or oral contraceptive pills. Oral northethisterone has also been recommended in dengue-associated hypermenorrhea [126]. Gynecologic practice for acute dysfunctional uterine bleeding in general has shifted away from oral hormonal therapy to tranexamic acid and mefanamic acid [127–129]. While the use of the latter in dengue is problematic, tranexamic acid may prove to be useful and awaits further research. Gastrointestinal bleeding in dengue always has the potential to be catastrophic because of its occult nature. In mild cases where no major hemorrhage is suspected, the use of proton pump inhibitors may have a favorable risk-to-benefit ratio. There is no evidence that platelet transfusion as primary prophylaxis in the absence of bleeding independently reduces the risk of bleeding or other severe outcomes [130–132].

Where bleeding is persistent, the administration of platelets may be considered, despite the transience of the effect on platelet levels. However, if blood loss is significant, administration of packed red cells or fresh whole blood is more urgent. Evidence for bleeding may be complicated to assess where bleeding is occult. Watching hemoglobin levels in isolation may give an incomplete picture because of the dynamic fluid shifts in dengue especially during the critical phase. Close watch of the hematocrit, clinical fluid accumulation, fluid balance, and hemodynamic status will provide a more complete picture to determine if the management of bleeding is necessary. Differentiating shock because of hemoconcentration and hemorrhagic shock is critical as the management is fundamentally different. Where bleeding remains uncontrolled, activated recombinant factor VII has been used with success [133, 134].

Thrombocytopenia itself has been used as a surrogate for impact on dengue progression in various therapeutic trials without any agents identified as successful in significantly improving platelet recovery in acute infection, as in, for example, high-dose intravenous immunoglobulin [81]. In a few cases, however, unusually persistent thrombocytopenia lasting far beyond the usual convalescent recovery period has been successfully treated with corticosteroids [135, 136].

Finally, there have been increasing reports of dengue-associated hemophagocytic lymphohistiocytosis [137–142] worryingly often associated with death or identified post-mortem. Prompt diagnosis is difficult given the need for biopsy and the complexity of the diagnostic criteria, and treatment may include corticosteroids or intravenous immunoglobulin in milder cases, to the use of cytotoxic and immunomodulatory agents under several trial protocols [143].

### Hepatic manifestations

While dengue-associated liver inflammation tends to be ubiquitous but transient, not requiring specific therapy [99], there are cases of acute liver failure likely due to a combination of direct viral cytopathic effects, liver ischemia through hemodynamic instability, and immunopathologic sequelae of infection [144]. Apart from supportive measures, a number of studies have promoted the use of N-acetylcysteine therapy in dengue-associated acute liver failure [145–147]. There has also been one case report of successful use of the molecular adsorbent recirculating system in dengue hemorrhagic fever [148].

### Respiratory manifestations

Dengue shock can be associated with respiratory compromise as recognized in the WHO 2009 guidelines [52] often associated with pleural effusion. In the absence of dengue-specific guidelines, use of standard intensive care protocols can be considered. One study recommended nasal continuous positive airway pressure rather than oxygen mask treatment in dengue patients with respiratory failure [149]. Pleural effusion can be the result of over-hydration leading to extravasation and third-space accumulation of fluid. Judicious fluid administration and frequent monitoring is critical to prevent this.

### Cardiac manifestations

Three major syndromic cardiac manifestations of dengue infection are myocardial impairment, arrythmias, and myocarditis [150•]. Apart from bradycardia, which is common in dengue [151], subtle signs of dysfunction may only be reflected in poorer than expected cardiovascular response to fluid infusion, which is hard to quantify. However, in a Vietnamese case series of 79 pediatric dengue patients uniformly subjected to echocardiography, over half showed signs of myocardial impairment measured by preload-independent indices such as velocities [152]. In an Indian case series of 67 children with severe dengue, 70 % had abnormalities in the Tei index compared with 48 % by ejection fraction and 37 % using the ratio of transmitral Doppler early filling velocity to tissue Doppler early diastolic mitral annular velocity (E/E) while being mostly asymptomatic [153]. As with other organ systems, pathogenesis is likely due to a combination of direct viral cytopathic effects, metabolic derangement, cytokine-mediated immunopathology, interstitial edema, and microvascular changes [150•]. Again, while no dengue-specific protocols have been proposed, the use of standard cardiology regimens has proven successful in case reports [154].

## Conclusion

The impact of dengue has the potential for severe morbidity in young and healthy individuals, especially in situations of massive outbreaks, as have been happening, for example, in Lahore, Pakistan in 2011, with 500,000 notified cases in a city of 5 million, and a dengue IgG seropositivity rate of 67.9 % the following year in a city not previously endemic for dengue [155]. Physicians unfamiliar with dengue may at short notice have to deal with critically ill patients either in an outbreak or as a result of travel-related dengue infection [156]. Management experience in severe dengue has largely occurred in the context of pediatric cases. Because of demographic transitions, climatico-ecologic change, and epidemiologic trends, adults are increasingly affected and thus we may have to focus on ensuring guidelines remain relevant or updated [157]. Increasingly, older adults are seen to be at risk from dengue-related mortality [63, 158]. A broad range of virologic, immunologic, clinical, and implementation science research will be required to combat this emerging pandemic.
